# Infection and the use of personal protective equipment among Primary Health Care workers during the COVID-19 pandemic

**DOI:** 10.1590/1518-8345.6870.4290

**Published:** 2024-08-12

**Authors:** Jarbas da Silva Ziani, Jenifer Härter, Bruna Lixinski Zuge, Debora da Cruz Payão Pellegrini, Josefine Busanello, Karlo Henrique dos Santos Herrera

**Affiliations:** 1 Universidade Federal de Santa Maria, Centro de Ciências da Saúde, Santa Maria, RS, Brazil.; 2 Universidade Federal do Pampa, Centro de Ciências da Saúde, Uruguaiana, RS, Brazil.; 3 Universidade Federal do Pampa, Centro de Ciências da Saúde Animal, Uruguaiana, RS, Brazil.

**Keywords:** Personal Protective Equipment, Covid-19, Health Personnel, Primary Health Care, Pandemics, Occupational Health

## Abstract

**Objective:**

to analyze the frequency and associated risk factors for COVID-19 infection and the availability of Personal Protective Equipment used by primary healthcare workers.

**Method:**

a cross-sectional study was conducted over six months in Rio Grande do Sul. Descriptive analysis was performed, with the comparison of independent samples using Pearson’s Chi-square test and Fisher’s Exact test (*p*<.05).

**Results:**

the study included 206 (27%) healthcare workers who presented COVID-19 symptoms. There was a statistical association for the following variables: availability of surgical masks (*p*=.003), seeking information on the correct use of personal protective equipment (*p*=.045), having attended people with flu-like syndrome (*p*=.024), and believing that the highest risk of contamination is when attending a patient positive for coronavirus disease (*p*=.001).

**Conclusion:**

the availability of personal protective equipment is indispensable for COVID-19 prevention, with special emphasis on the use of surgical masks. Furthermore, the study highlighted the importance of providing Personal Protective Equipment in conjunction with guidance on its use.

## Highlights

**(1)** Highlighted impacts on the distribution of PPE necessary for worker safety.

**(2)** Emphasized the need for training and education regarding the use of PPE.

**(3)** Found significance regarding the availability of surgical masks.

**(4)** Identified the need for further research on health safety topics.

**(5)** Revealed a high incidence of symptomatic workers and positive cases of COVID-19.

## Introduction

The coronavirus disease (COVID-19) pandemic has become an emblematic challenge for the world, as it has been challenging the healthcare systems of affected countries since its onset^1^. Considering its rapid spread^1^ and the alarming official statistics, updated data showed that by April 2023, the world had recorded 6,908,554 deaths, with 700,811 of these in the Brazilian territory^2^.

Although new viral mutations and adaptations continue to occur, it is estimated that a person infected with the Severe Acute Respiratory Syndrome Coronavirus 2 (SARS-CoV-2) has the potential to infect between 1.5 to 3.5 other individuals^3^. Healthcare workers have a probability of infection up to three times higher when compared to the general population, as they are constantly exposed to contact with patients infected with the virus during their work routine^4^ ^)-(^ ^5^.

Analyzing the number of cases reported worldwide, it has been noted that 15% represent infections in healthcare workers^6^. In July 2021, during the peak of the pandemic in Brazil, there were 120,240 (27.1%) cases of healthcare workers infected by the virus and 549 (96.2%) deaths, which translates to one death every 17 hours during that period^7^. In this scenario, Primary Health Care (PHC) proved essential in combating the pandemic, accommodating up to 80% of the total mild and moderate cases of symptomatic individuals^8^.

Family Health Teams (FHTs), acting in their territories, play a fundamental role in the care assistance network, being crucial for addressing any epidemic^9^. Therefore, for care to be provided safely, the use of Personal Protective Equipment (PPE) during assistance was necessary. One study showed that 85% of healthcare workers who used PPE during their care, especially the N95 mask, did not contract COVID-19^10^. 

Accordingly, the duty of healthcare institutions to provide their workers with the necessary PPE and to provide proper guidance for its correct use is emphasized^11^. This equipment serves as an individual mechanical barrier against pathogens, minimizing healthcare workers’ exposure. The most commonly used equipment for caring for COVID-19 patients were masks, gloves, disposable gowns, caps, gowns, face shields, protective goggles, NIOSH-certified particulate respirators (N95, N99, and N100), and Filtering Facepiece Respirators (FFP1, FFP2, or FFP3)^11^.

In recent years, the field of occupational health and safety has established intervention measures aimed at promoting protection, promotion, recovery, rehabilitation of health, and worker safety, through the creation of policies, programs, and practices that seek well-being^12^. The health of these workers faced unprecedented occupational risks of morbidity and mortality, related to aspects considered harmful to safety, mainly caused by the lack of PPE, associated with exposure to infected patients, workload overload, and lack of infection control, aspects considered harmful to the safety of healthcare providers^13^.

As evidenced by a Korean study, there was a significant increase in the workload of healthcare workers, as they not only provided specific care and assistance to infected patients but also began to perform the cleaning of the environments. This, combined with a shortage of workers, medical supplies, and PPE, resulted in poor working conditions for these healthcare providers^14^.

Throughout the COVID-19 pandemic, countries were affected by shortages in the global production and supply chain, high prices, and problems with product quality, making timely material delivery difficult and hindering healthcare institutions from maintaining adequate stock to ensure the protection of healthcare workers^15^. In this regard, the fundamental role of the government in addressing this situation is emphasized, as it is responsible for coordinating efforts to ensure that the hardest-hit geographic areas have the necessary equipment^15^.

Considering this, the present study is justified by the need for reflections and research regarding the quality of work of PHC workers exposed to the changes caused by the COVID-19 pandemic, as studies predominantly focus on aspects related to workers in hospital care. It is also emphasized that it is of paramount importance that these workers receive greater visibility, as they constitute the front line of PHC, which is the main entry point and screening for symptomatic COVID-19 patients in health services amid the pandemic containment efforts.

The study’s results may assist healthcare workers, especially nurses, with information for healthcare and nursing practice, enabling reflection on the constant need for biosafety protocols and the use of personal protective equipment in healthcare routines. Therefore, this study aimed to analyze the frequency and associated risk factors with COVID-19 infection and the availability of Personal Protective Equipment used by PHC workers.

## Method

### Study design

This was a cross-sectional study conducted with healthcare workers. In the organization of the text, the recommendations of the Checklist for Reporting Results of Internet E-Surveys guidelines and Strengthening the Reporting of Observational Studies in Epidemiology (STROBE) for cross-sectional studies were followed^16^.

### Study setting

The study was conducted in the municipality of Uruguaiana, Rio Grande do Sul-RS, Brazil. The city holds significant commercial and international strategic interest, as it is equidistantly located from the capitals Porto Alegre, Montevideo, Buenos Aires and Asunción^17^. Regarding the number of COVID-19 cases, the city had recorded 155,362 infections and 637 deaths at the time of data collection^18^.

The healthcare coverage in the municipality by Primary Health Care teams is estimated at 66%, as it has 19 health units with 23 Family Health Teams (FHTs) in the urban area and one Rural Family Health Team. Additionally, there is a mobile Health Team that serves users from rural areas of the municipality not covered by local health units^19^.

### Study population

The study population consisted of all healthcare workers (physicians, nurses, nursing technicians and assistants, dentists, pharmacists, pharmacy assistants, physiotherapists, nutritionists, speech therapists, biomedical professionals, social workers, physical educators, veterinarians, psychologists, administrative staff, hygienists and receptionists) affiliated with the Municipal Health Department (*N*=750), who were followed for six months from March to August 2020. No sample calculation was performed since all employees were included for monitoring, and at the time, there were no studies estimating the proportion of symptomatic healthcare workers.

The inclusion criterion considered workers who were active during the COVID-19 pandemic period. Exclusion criteria included workers hospitalized due to symptoms before the scheduled testing date at the Municipal Health Department and unable to respond to the questionnaire.

Workers were monitored by the study and considered suspected cases of COVID-19, with an indication for diagnostic testing, if they presented symptoms and clinical signs compatible with influenza-like illness, such as fever, cough, runny nose, sore throat, headache, vomiting, diarrhea, dyspnea, and general malaise. They were also tested and evaluated if they shared workspace with another symptomatic professional who tested positive, or if they were absent from work due to symptoms, notified by Short Message Service (SMS) or under home isolation due to clinical recommendation, with a doctor’s request for COVID-19 testing. Accordingly, 206 healthcare workers who at some point during the monitoring period presented characteristic symptoms for COVID-19 were included in the study.

Monitoring was conducted through telephone contact, initiated after notification of symptoms by SMS. Healthcare workers received a questionnaire after the first test, regardless of the first test result. Throughout the monitoring, with each new episode of symptoms reported by the workers, retesting for COVID-19 was performed. For subsequent tests after the first one, data were collected again via a form only in cases of positive results.

### Measuring instrument

The applied form consisted mainly of closed-ended questions and sought to collect information relevant to demographic and socioeconomic characteristics, flu-like symptoms, and the timing of their onset. It also contained information regarding the workplace, the number of hours spent attending patients, and knowledge, use, and availability of PPE. The form was developed by the research authors based on the scientific literature and their inquiries about the topic. After creation, the form was sent to a committee of five healthcare experts for content evaluation. After the recommended adjustments, the final version was defined following all suggestions.

Subsequently, an online form was created using the Google Forms platform to facilitate participant access, especially during a pandemic period, as it can be accessed from any desktop computer and mobile device, covering all operating systems as long as there is internet access. The creation of the form took into account a quick survey conducted by the service managers regarding the availability of these resources for healthcare workers. The form was then subjected to a pilot test with 15 members of the research group who were healthcare workers, excluded from the analyzed sample. The purpose of the pilot test was to analyze whether the items were easy to understand and appropriate to be responded to through an online survey.

Regarding the COVID-19 testing of the healthcare workers, the MedTeste Coronavirus (COVID-19) rapid tests for immunoglobulin G (IgG)/immunoglobulin M (IgM) were used in the first week of the study. The rapid testing was conducted between the 10^th^ and 12^th^ day of symptoms. Following new testing determinations, the Reverse Transcriptase followed by Polymerase Chain Reaction (RT-PCR) test, provided by the Ministry of Health during the pandemic, was used. The RT-PCR test was collected between the 4^th^ and 8^th^ days of symptoms in the workers. The tests used in the study were collected once for each symptomatic episode of the worker.

### Study variables

The quantitative variables were age, years of education and days of work, which characterized the sample. The sample was also stratified into two groups, one group of workers who tested negative (NG) and another composed of those who tested positive (PG). The healthcare worker who presented a positive result, regardless of the number of tests conducted, was included in the positive group. Therefore, the negative group refers to not having tested positive in any monitoring test throughout the study period.

The categorical variables analyzed were: a) dichotomous (yes/no): pulmonary diseases, cardiovascular diseases, receipt of PPE, received guidance on the use of PPE, sought information, treated flu-like syndrome, treated COVID-19 cases and sex (male/female); b) nominal: level of education (higher/high school) and people’s access to the workplace (limited/restricted/direct care), self-assessment of knowledge about PPEs (good/excellent/average/very poor), opinion regarding the highest risk moment for the worker regarding PPE use (when dressing/when removing gloves for hand hygiene/only when attending suspected cases/when removing the gown/when leaving/other), for this variable, the responses with low occurrence were grouped into the “other” category. 

The receipt of PPE and the availability of each piece of equipment: cap, protective goggles, gloves, face shield, disposable gown, N95 or PFF mask, surgical mask, and cloth mask, were also analyzed.

### Data collection

Data collection took place from May to December 2020, with up to three attempts to contact participants via phone calls for inclusion in the study. The form was sent after workers manifested symptoms and confirmed interest in participating or remaining in the study.

After contact, the COVID-19 testing or sample collection occurred at the outpatient clinic of the municipal polyclinic or at the triage centers set up for pandemic response in the municipality. Transportation and analysis of the samples were carried out by the *Laboratório Central do Rio Grande do Sul* (LACEN-RS). Upon receipt of the report from LACEN to the Municipal Health Department, the research team was notified and then phone contacts were made. During the phone call, the result was communicated, and researchers provided relevant care instructions based on the condition presented by each healthcare worker. After their acceptance to participate in the study, the form was sent. It should be emphasized that the creation of the online form and phone contact were intended to promote safety and avoid subjecting the individuals involved to risk.

### Data analysis

Responses to the forms were obtained from the cloud data report in an Excel Office spreadsheet available through the platform used. Data completion was reviewed, and control questions were checked for data quality analysis. One case was considered a loss to follow-up, as they did not complete the form at the second testing, leaving only their first testing and corresponding form in the sample. Subsequently, the data were transferred to the Statistical Package for the Social Sciences (SPSS) version 25.0 software, in which descriptive analysis, mean and standard deviation (*SD*) (±).

Furthermore, independent samples were performed through Pearson’s Chi-square test or Fisher’s Exact test, assuming association when the *p*-value was less than .05 and confidence intervals of 95%.

### Ethical aspects

The study was approved by the Research Ethics Committee under authorization number 30837420.0.0000.5323 and followed all ethical guidelines of Resolution 466/12 of the National Health Council. The consent form was made available online. It should be emphasized that the questionnaire only became available to be completed after the participant agreed to participate by selecting the option “I have read and understood the research and I want to participate” on the consent form.

## Results

Of the 750 healthcare workers monitored, 206 who presented flu-like symptoms in the first six months of the pandemic participated in this research, corresponding to 27% of the total number of healthcare workers monitored. Among those tested, 70 (34.0%) tested positive at some point during the six-month follow-up. The mean age was 39 years (*SD*±11), with a minimum age of 19 years and a maximum of 81 years. The mean number of years of education was 10 (*SD*±9), with a minimum of one year and a maximum of 29 years. Regarding the characteristics of the work schedule, the mean number of active days per week was five (*SD*±1), with seven hours per day (*SD*±3).

The majority of participants in the study were female, 156 (75.72%), and 50 (24.28%) were male. 182 (88.34%) had no pulmonary problems and 164 (79.61%) had no cardiovascular alterations. A total of 143 (69.41%) were workers with higher education and 161 (78.15%) provided direct patient care. Table 1 displays the characteristics of the healthcare workers according to the test results.

**Table 1:** Characterization of the participants according to COVID-19 test results (*n* = 206). Uruguaiana, RS, Brazil, 2020

Variables	NG* 136 (66.0%)	PG^‡^ 70 (34.0%
	***n* (%)** ^†^	***n* (%)** ^†^
**Sex**		
Female	107 (78.7)	49 (70.0)
Male	29 (21.3)	21 (30.0)
**Pulmonary disease**		
No	117 (86.0)	65 (92.9)
Yes	19 (14.0)	5 (7.1)
**Cardiovascular disease**		
No	111 (81.6)	53 (75.7)
Yes	25 (18.4)	17 (24.3)
**Education level**		
High school	41 (30.1)	22 (31.4)
Higher education	95 (69.9)	48 (68.6)
**Work sector**		
Restricted access	2 (1.5)	0 (0.0)
Limited access	24 (17.6)	19 (27.1)
Direct care	110 (80.9)	51 (72.9)

*NG = Negative Group; ^‡^PG = Positive Group; ^†^Absolute number and (%)

It was identified that, among all participants, 197 (95.63%) workers received some type of PPE, while 9 (4.37%) remained unassisted in terms of receiving it. Regarding receiving guidance for the use of this equipment, 144 (69.90%) stated they received some type of information and 146 (70.87%) actively sought information on its use. In terms of the perception of knowledge about the use of PPE, 152 (73.78%) expressed good knowledge, followed by 27 (13.10%) excellent, 25 (12.13%) average and 2 (0.97%) very poor.

When examining the types of PPE that the participants received, 7 (3.39%) stated they received caps, 12 (5.82%) protective glasses, 147 (71.35%) gloves, 145 (71.35%) face shields, 159 (77.18%) disposable gowns, 140 (67.96%) N95 or PFF2 masks, 162 (78.64%) surgical masks and 115 (55.82%) cloth masks. It should be highlighted that some healthcare workers received more than one type of PPE.

In the analysis of patient care, it was found that 165 (80.9%) healthcare workers attended individuals presenting symptoms characteristic of influenza-like illness at some point, and 173 (83.98%) reported having attended individuals who tested positive for COVID-19. Considering the aforementioned results, Table 2 shows that there was adequate availability of PPE for the healthcare workers.

**Table 2:** Availability and knowledge about PPE* among healthcare workers in COVID-19 care according to the test results (n = 206). Uruguaiana, RS, Brazil, 2020

Variables	NG^†^ 136 (66.0%)	PG^§^ 70 (34.0%)	*p* ^||^
	*n* (%)^‡^	*n* (%)^‡^	
Received PPE* ^¶^	132 (97.1)	65 (92.9)	.963
Received guidance on PPE* use ^¶^	94 (69.1)	50 (71.4)	.134
**Sought information** ^¶^	116 (85.3)	54 (77.1)	.045
**Attended patients with flu-like symptoms** ^¶^	110 (80.9)	55 (78.6)	.024
**Attended COVID-19 cases** ^¶^	112 (82.4)	61 (87.1)	.138
**Risk of contagion during dressing**			
During removal of PPE*^¶^	75 (55.1)	45 (64.3)	.084
Only when attending suspected cases^¶^	34 (25.0)	7 (10.0)	.001
Others^¶^	27 (19.9)	18 (25.7)	.256
PPE* received			
Cap**	4 (2.9)	3 (4.3)	.265
Protective glasses**	8 (5.9)	4 (5.7)	1.256
Gloves^¶^	99 (72.8)	48 (68.6)	.337
Face shield^¶^	91 (66.9)	54 (77.1)	.281
Disposable gown^¶^	103 (75.7)	56 (80.0)	.212
N95 mask^¶^	89 (65.4)	51 (72.9)	.707
Surgical mask^¶^	113 (83.1)	49 (70.0)	.003
Cloth mask^¶^	72 (52.9)	43 (61.4)	.897

*PPE = Personal Protective Equipment; ^†^NG = Negative Group; ^§^PG = Positive Group; ^‡^ *n* (%) = Absolute number and (%)^; ||^ *p* = Level of significance (*p*<.05); ^¶^Pearson Chi-square; ^**^Fisher’s Exact Test

Statistical association was identified in the Pearson Chi-square analysis regarding the test result and the surgical mask availability (*p*=.003) variable. Additionally, concerning the participants’ knowledge about the highest risk moment for disease transmission (*p*=.001), there was a predominance of workers who considered risk only when attending suspected COVID-19 cases. Furthermore, an association was noted with participants who mentioned autonomously seeking information about the correct use of PPE (*p*=.045). Also, attending individuals with flu-like symptoms was associated with testing positive for COVID-19 (*p*=.024). Regarding the classification of healthcare workers’ knowledge about PPEs, in the NG, 16 (11.8%) rated their knowledge as excellent, 99 (72.8%) as good, 19 (14.0%) as moderate and 2 (1.5%) as very poor. In the PG, 11 (15.7%) rated their knowledge as excellent, 53 (75.7%) as good and 6 (8.6%) as moderate, with no participant in this group rating their knowledge as very poor.

## Discussion

Personal Protective Equipment constitutes unquestionably fundamental safety items to promote the protection of healthcare workers, aiming to minimize direct exposure to the SARS-CoV-2 virus. According to the data from this study, it is noted that not all healthcare workers received PPE, making a portion of these workers even more susceptible to infection. 

Accordingly, attention is drawn to the importance of minimizing the occupational risks faced by healthcare workers and the obligation of institutions to provide this equipment, as it is the first step in ensuring the health and safety of this population, as healthcare workers were considered the most valuable resource of each country, faced with the impossibility of rapidly replacing professionals experienced in the pandemic response^20^. 

Regarding the discrepancy in access to PPE in each country, especially when analyzing availability for Primary Health Care (PHC), it was observed that in countries with lower market power, such as Brazil, healthcare workers experienced problems regarding the scarcity and low quality of this equipment; meanwhile, countries with higher monetary conditions had access to extra protection equipment associated with high engineering technology, preserving the health of their workforce^21^. 

Comparing the receipt of PPE between the groups that tested positive for COVID-19 and those that tested negative, it was identified that the receipt of surgical masks was related to the group of workers who tested negative. That is, receiving and using it is considered a factor of extremely high impact for reducing COVID-19 transmission, a result that corroborates a study published before the COVID-19 pandemic, which considered updates on Severe Acute Respiratory Syndrome (SARS). 

Therefore, the high effectiveness of surgical masks in reducing virus transmission is ensured^22^ ^)-(^ ^23^. 

Other studies with healthcare workers have shown that using PPE during clinical practice is a challenging task and may have harmful effects on health, such as neurological and physical dysfunctions, manifested by headache, irritability, difficulty in decision-making, nausea and shortness of breath^24^ ^)-(^ ^25^. These effects were associated with organic reactions such as hypoxia and hypercapnia, environmental factors such as working in enclosed spaces, high temperatures, humidity, and also stressors caused by the pandemic itself^24^ ^)-(^ ^25^. 

However, it is plausible to state that the benefits outweigh the risks, justifying correct adherence to the use of PPE in work practices. The use of masks by healthcare workers is extremely relevant in the study, reinforcing the importance of being implemented at all stages of patient care seeking health services with symptoms compatible with respiratory syndrome, from their arrival, screening and waiting, to discharge/transfer or death^26^. Such behavior should surpass the pandemic period and be considered a daily protocol of health services in caring for patients with respiratory symptoms compatible with viral diseases.

Furthermore, it is necessary to emphasize that cloth masks, although widely used for population protection at the beginning of the pandemic, are not recommended for use by healthcare workers, as they are not considered standard PPE mainly due to the lack of evidence of their effectiveness against SARS-CoV-2 transmission^23^. Nonetheless, the study highlights the significant number of healthcare workers who received cloth masks to use in their workplaces, of which 43 (61.4%) tested positive for COVID-19 at some point during the follow-up period. 

It is hypothesized that the factors that led to the choice of cloth masks by healthcare workers were the scarcity of surgical masks, these being insufficient to meet the demand for use in the context of analysis. However, recommendations^27^ already warned that this mask should be the last resort used in healthcare assistance to any patient, whether respiratory symptomatic or not. Moreover, the guidance also stated that in the absence of N95 or PFF2 masks for replacement within the indicated period, the worker should use them beyond the expiration date indicated by the producers, although it was emphasized that there was no guarantee of effectiveness under these conditions^27^. This may have significantly contributed to the high frequency of cases among healthcare workers who tested positive for COVID-19 in the study.

In addition to the cost-effectiveness and durability due to their reuse, another factor that reinforced the use of cloth masks in the general population was the need to prioritize the supply for healthcare workers. Results from an American study discussing the prioritization of this supply among healthcare workers showed that only 1/3 (33%) of nursing professionals had access to N95 masks, while for the medical team, it totaled 100%^28^. 

It is emphasized that to ensure the effectiveness of PPE, they need to be used correctly and the indications recommended by the manufacturers followed. Therefore, it is necessary to conduct frequent training for healthcare workers, a circumstance reiterated by the data from this study, where participating professionals who attended people with flu-like symptoms tested positive for COVID-19 at some point in the study (*p*=.024). Furthermore, one in five professionals believed that the moment of greatest risk of infection was only when attending users identified as suspects. 

Consequently, the importance of training in dressing and undressing issues is clear, including notions about disease attenuation and handling of suspected or confirmed users^29^. Studies confirm that workers who use PPE correctly and are attentive to the indicated time of use are less susceptible to COVID-19 infection, increasing their sense of security^29^ ^)-(^ ^30^.

Furthermore, in line with the aforementioned statements, it was observed that study participants who indicated they sought information autonomously about the correct use of PPE were less likely to be infected with COVID-19. Corroborating the findings, a study revealed that knowledge about the correct handling of PPE is directly related to protective factors^31^.

The implementation of service protocols, as well as training for teams, is fundamental for adherence to correct and necessary dressing care to minimize the occurrence of infections by SARS-CoV-2 in healthcare workers. Therefore, its use was indispensable during the pandemic and it is essential to structure the logistics of supplying these inputs, as well as to organize and implement a plan that promotes the proper and rational use of this PPE^32^, even in the face of sporadic cases or post-pandemic outbreaks.

Likewise, it is suggested that the high rate of infected healthcare workers may have been influenced by improper handling or timing in the use, involving putting on and removing PPE. This was evidenced by the 62 (30.1%) participants who reported that they did not receive guidance on the correct use of the equipment. From this point of view, a study showed that when healthcare workers use PPE and are well-oriented and updated regarding its purpose, they have a lower probability of being infected with the SARS-CoV-2 virus^10^.

It should also be considered that, in the pandemic scenario, many healthcare workers needed to be hired or reassigned from their routine functions, seeking to meet the needs of healthcare services in the face of critical moments caused by the high number of people and healthcare workers infected, conditions that required immediate learning^10^ ^,^ ^33^ ^-^ ^34^. This fact reinforces, once again, the importance of implementing instruments and training to provide basic notions of healthcare routine regarding workplace protection^33^.

Among the study limitations, it was considered that data regarding the full-time use of PPE in the work routine or the frequency of replacement by the professional were not included, nor was the scarcity of supplies at any time investigated. Another aspect that requires attention is the use of the rapid test only in the first week of the study, applied to nine participants. The change occurred due to the publication of a state technical note. Considering the monitoring method, the guideline for the exclusive use of RT-PCR for testing in the study followed the reality of the context under analysis.

It should be mentioned that the results found through this study in the context of COVID-19 highlighted the need for research on the health safety of PHC workers regarding exposure to infectious diseases since the scientific literature emphasizes the confrontation in the hospital environment. In addition to the elaboration of pertinent actions for the field of health education, in order to guide healthcare workers about the importance of using PPE in their work practices, as important as providing the equipment, is that they are used correctly and as indicated.

## Conclusion

The study identified a high frequency of symptomatic healthcare workers testing positive for COVID-19. Furthermore, the results clearly indicate that the distribution, use, and guidance on the correct management of PPE in the healthcare workers’ practice is indispensable for preventing COVID-19, with special emphasis on the use of surgical masks.

Consequently, this study demonstrated a weakness in service management by assuming that healthcare workers have sufficient knowledge about the use of PPE in their daily work. The provision of PPE alone cannot be considered sufficient without proper guidance on its use. Therefore, the results indicated the need for appreciation and guidance for the effective protection of nursing and PHC workers.
