# Supplementary material for: Infection and the use of personal protective equipment among Primary Health Care workers during the COVID-19 pandemic
Source: Rev Lat Am Enfermagem. 2024 Aug 12;32:e4290. doi: 10.1590/1518-8345.6870.4290 (PMC11321185; doi:10.1590/1518-8345.6870.4290)
Supplement: Translation

# Translation: Infecção e uso de equipamentos de proteção individual entre trabalhadores da Atenção Primária à Saúde durante a pandemia da COVID-19

**DOI:** 10.1590/1518-8345.6870.4291

**Authors:** Jarbas da Silva Ziani, Jenifer Härter, Bruna Lixinski Zuge, Debora da Cruz Payão Pellegrini, Josefine Busanello, Karlo Henrique dos Santos Herrera

**Affiliations:** 1 Universidade Federal de Santa Maria, Centro de Ciências da Saúde, Santa Maria, RS, Brasil.; 2 Universidade Federal do Pampa, Centro de Ciências da Saúde, Uruguaiana, RS, Brasil.; 3 Universidade Federal do Pampa, Centro de Ciências da Saúde Animal, Uruguaiana, RS, Brasil.

**Keywords:** Equipamento de Proteção Individual, Covid-19, Pessoal de Saúde, Atenção Primária à Saúde, Pandemias, Saúde Ocupacional

## Abstract

**Objetivo:**

analisar a frequência e os fatores de risco associados à infecção por COVID-19 e a disponibilidade de Equipamentos de Proteção Individual utilizados por trabalhadores da atenção primária à saúde.

**Método:**

estudo transversal, com duração de seis meses, realizado no Rio Grande do Sul. Para a análise, realizou-se a análise descritiva, com comparação de amostras independentes pelo teste de Qui-quadrado de Pearson e Exato de Fisher (p<0,05).

**Resultados:**

participaram do estudo 206 (27%) trabalhadores da saúde que apresentaram sintomas de COVID-19. Verificou-se associação estatística para as variáveis disponibilidade da máscara cirúrgica (p=0,003), buscar informações sobre o uso correto dos equipamentos de proteção individual (p=0,045), ter atendido pessoas com síndrome gripal (p=0,024) e acreditar que o maior risco para contaminação é atender um paciente positivo para *coronavirus disease* (p=0,001).

**Conclusão:**

a disponibilidade de equipamentos de proteção individual é indispensável para prevenção contra a COVID-19, com especial ênfase na utilização da máscara cirúrgica. Além disso, o estudo apontou a importância de fornecer os Equipamentos de Proteção Individual de forma articulada com a orientação assistencial para o seu uso.

## Destaques

**(1)** Apontou-se impactos na distribuição de EPI necessário para a segurança do trabalhador.

**(2)** Destacou-se a necessidade de capacitações e treinamentos acerca da utilização dos EPI.

**(3)** Houve significância quanto à disponibilidade da máscara cirúrgica.

**(4)** Apontou-se a necessidade da realização de pesquisas acerca do tema da segurança em saúde.

**(5)** Evidenciou-se alta incidência de trabalhadores sintomáticos e positivos para COVID-19.

## Introdução

A pandemia do *coronavirus disease* (COVID-19) tornou-se uma emblemática indagação para o mundo, pois vem desafiando os sistemas de saúde dos países afetados desde o seu início^1^. Tendo em vista seu caráter de disseminação acelerada^1^ e as estatísticas oficiais que foram alarmantes, dados atualizados mostraram que, em abril de 2023, o mundo já contava com 6.908.554 mortes, dessas, 700.811 em solo brasileiro^2^.

Embora ainda exista a ocorrência de novas mutações e adaptações virais, estima-se que uma pessoa contaminada pelo *Severe Acute Respiratory Syndrome Coronavirus* 2 (SARS-CoV-2) possui a possibilidade de infectar entre 1,5 a 3,5 outras pessoas^3^. Os trabalhadores da saúde possuem uma probabilidade de infecção de até três vezes mais quando comparados à população em geral, uma vez que, em sua rotina de trabalho, estão constantemente expostos ao contato com pacientes infectados pelo vírus^4^ ^)-(^ ^5^. 

Ao analisar o número de casos registrados no mundo, nota-se que 15% representam a infecção em trabalhadores da saúde^6^. Em julho de 2021, durante o pico da pandemia no Brasil, registraram-se 120.240 (27,1%) casos de trabalhadores da saúde infectados pelo vírus e 549 (96,2%) óbitos, representando uma morte ocorrida a cada 17 horas naquele período^7^. Diante desse cenário, a Atenção Primária à Saúde (APS) mostrou-se imprescindível no combate da pandemia, acolhendo até 80% do total de casos leves e moderados dos sintomáticos^8^.

As Equipes de Saúde da Família (ESF), na atuação em seus territórios, possuem papel fundamental na rede assistencial de cuidados, sendo primordiais para o enfrentamento de qualquer epidemia^9^. Dessa forma, para os atendimentos serem realizados seguramente, foi necessária a utilização dos Equipamentos de Proteção Individual (EPI) durante a assistência. Um estudo evidenciou que 85% dos trabalhadores da saúde que usaram EPI durante os seus atendimentos, em especial o uso da máscara N95, não contraíram a COVID-19^10^. 

Nesse sentido, ressalta-se o dever das instituições de saúde de fornecer aos seus trabalhadores os EPI necessários, bem como fornecer as devidas orientações para o correto uso^11^. Tais equipamentos servem como uma barreira mecânica individual contra agentes patógenos, minimizando a exposição dos trabalhadores da saúde. Os mais utilizados para o atendimento a pacientes com COVID-19 foram as máscaras, luvas, aventais descartáveis, toucas, capotes, protetores faciais, óculos de proteção e respirador particulado do tipo NIOSH-*certified* (N95, N99 e N100) e as Peças Faciais Filtrantes (PFF1, PFF2 ou PFF3)^11^. 

Nos últimos anos, o campo da saúde e da segurança do trabalhador estabeleceu medidas de intervenções destinadas a promover proteção, promoção, recuperação, reabilitação da saúde e da segurança do trabalhador, por meio da criação de políticas, programas e práticas que buscassem o bem-estar^12^. A saúde desses trabalhadores enfrentou riscos ocupacionais sem precedentes de morbidade e mortalidade, relacionados aos aspectos considerados prejudiciais à segurança, ocasionados principalmente pela falta de EPI, associada à exposição aos pacientes infectados, à sobrecarga de trabalho e à falta de controle de infecções, aspectos considerados prejudiciais à segurança dos profissionais da saúde^13^.

Conforme evidenciado por estudo coreano, houve um aumento expressivo na carga de trabalho de trabalhadores da saúde, visto que esses, além de prestar cuidados e atendimentos específicos aos pacientes infectados, passaram a realizar também a higiene dos ambientes. Isso, associado com uma escassez de trabalhadores, de suprimentos médicos e EPI, resultou em condições precárias de trabalho para esses trabalhadores da saúde^14^. 

Em diversos momentos da pandemia da COVID-19, os países foram afetados pela escassez na cadeia de produção e de abastecimento global, preços elevados e problemas com a qualidade dos produtos. Da mesma forma a entrega de materiais em tempo hábil foi prejudicada, dificultando que as instituições de saúde mantivessem o estoque para garantir a proteção dos trabalhadores da saúde^15^. Nesse sentido, ressalta-se o papel fundamental do governo frente a essa situação, uma vez que ele detém a responsabilidade por coordenar esforços para garantir que as áreas geográficas mais atingidas possuam os equipamentos necessários^15^. 

Visto isso, o presente estudo justifica-se pela necessidade de reflexões e pesquisas acerca da qualidade de trabalho dos trabalhadores da APS expostos às mudanças ocasionadas pela pandemia da COVID-19, uma vez que se evidenciou que estudos estão voltados predominantemente para aspectos relativos aos trabalhadores atuantes na atenção hospitalar. Ainda, salienta-se que é de suma importância que esses trabalhadores recebam maior visibilidade, visto que são eles que constituem a linha de frente da APS, que se caracteriza como a principal porta de entrada e triagem dos pacientes sintomáticos da COVID-19 nos serviços de saúde frente ao apaziguamento da pandemia. 

Os resultados do estudo poderão auxiliar os trabalhadores da saúde, em especial o enfermeiro, com informações para a prática em saúde e enfermagem, possibilitando uma reflexão acerca da necessidade na retomada constante sobre protocolos de biossegurança e uso de equipamentos de proteção individual na rotina de cuidados em saúde. Portanto, o objetivo deste manuscrito foi analisar a frequência e os fatores de risco associados à infecção por COVID-19 e a disponibilidade de Equipamentos de Proteção Individual utilizados por trabalhadores da APS.

## Método

### Delineamento do estudo

Trata-se de um estudo transversal, realizado com trabalhadores da saúde. Na organização do texto, foram seguidas as recomendações do *Checklist for Reporting Results of Internet E-Surveys guidelines and Strengthening the Reporting of Observational Studies in Epidemiology* (STROBE) para estudos transversais^16^.

### Cenário do estudo

O estudo foi realizado no município de Uruguaiana, Rio Grande do Sul-RS, Brasil. A cidade possui um importante interesse estratégico, comercial e internacional, tendo em vista que está localizada equidistantemente das capitais: Porto Alegre, Montevidéu, Buenos Aires e Assunção^17^. Quanto ao número de casos de COVID-19 a cidade, até o momento, registrou 155.362 infectados pela doença e desses, 637 evoluíram para óbito^18^.

A cobertura de saúde do município pelas equipes de Atenção Básica está estimada em 66%, uma vez que conta com 19 unidades de saúde, com 23 ESF no perímetro urbano e uma Equipe Saúde da Família Rural. Possui, ainda, uma equipe de Saúde itinerante, a qual atende os usuários das localidades rurais do município não contempladas com unidades de saúde locais^19^. 

### População do estudo

A população do estudo foi constituída pelo total de trabalhadores da saúde (médicos, enfermeiros, técnicos e auxiliares de enfermagem, dentistas, farmacêuticos, auxiliares de farmácia, fisioterapeutas, nutricionistas, fonoaudiólogos, biomédicos, serviço social, educador físico, médicos veterinários, psicólogos, servidores administrativos, higienistas e recepcionistas), todos vinculados à Secretaria Municipal de Saúde (N=750), os quais foram acompanhados durante seis meses, no período de março a agosto de 2020. Não foi realizado cálculo amostral, uma vez que a totalidade de servidores foi incluída para acompanhamento e, na época, não havia estudos que estimassem a proporção de trabalhadores da saúde sintomáticos.

Como critério de inclusão, consideraram-se trabalhadores que estivessem em atividade no período da pandemia da COVID-19. Quanto aos de exclusão, foram considerados trabalhadores em internação hospitalar em decorrência da sintomatologia antes da data de agendamento prevista para testagem na Secretaria Municipal de Saúde, inviabilizados de responder ao questionário. 

Os trabalhadores foram acompanhados pelo estudo e considerados caso suspeito de COVID-19, com indicação de testagem diagnóstica, ao apresentarem sintomas e sinais clínicos compatíveis com síndrome gripal, como febre, tosse, coriza, dor de garganta, cefaleia, vômito, diarreia, dispneia e mal estado geral. Também foram testados e avaliados em casos de: dividirem o espaço de trabalho com outro profissional sintomático que apresentou testagem positiva e, se afastado das atividades laborais em decorrência de sintomatologia, mediante notificação por *Short Message Service* (SMS) ou isolamento domiciliar por recomendação clínica, com indicação de teste para COVID-19 por solicitação médica. Dessa forma, foram incluídos no estudo 206 trabalhadores da saúde que, em algum momento durante o período de acompanhamento, apresentaram sintomatologia característica para COVID-19.

O acompanhamento ocorreu por meio de contato telefônico, realizado após a notificação de sintomatologia por SMS. Os trabalhadores da saúde receberam um questionário depois da primeira testagem, independente do resultado do primeiro teste. Ao longo do monitoramento, a cada novo episódio de sintomas relatados pelos trabalhadores, procedeu-se à retestagem para COVID-19, sendo que para os testes subsequentes ao primeiro, somente em casos de resultado positivo, coletaram-se novamente os dados via formulário. 

### Instrumento de medida

O formulário aplicado constituiu-se por um bloco de questões fechadas em sua maioria, e visou coletar informações pertinentes a características demográficas, socioeconômicas, sintomatologia gripal e temporalidade de seu início. Continha também informações referentes ao local de trabalho, número de horas em atendimento aos pacientes, conhecimento, uso e disponibilidade de EPI. O formulário foi elaborado pelos autores da pesquisa, com base na literatura científica e em seus questionamentos acerca do tema. Posteriormente à criação, encaminhou-se o formulário a um comitê de cinco especialistas na área da saúde para avaliação do conteúdo. Após os ajustes recomendados, definiu-se a versão final seguindo todas as sugestões. 

Logo, criou-se um formulário *online* na plataforma *Google Forms*, com o intuito de facilitar o acesso dos participantes, principalmente em um período de pandemia, já que pode ser acessada por quaisquer computadores de mesa e dispositivos móveis, contemplando todos os sistemas operacionais caso possua acesso à internet. Para a criação do formulário, considerou-se uma pesquisa rápida realizada pelos gestores dos serviços a respeito da disponibilidade desses recursos pelos trabalhadores de saúde. 

Por conseguinte, o formulário foi submetido ao teste-piloto com 15 integrantes do grupo de pesquisa que eram trabalhadores da saúde, excluídos da amostra analisada. O intuito do piloto foi analisar se os itens eram de fácil compreensão e adequados para serem respondidos, por meio de uma consulta *online*.

Já os testes utilizados para testagem para COVID-19 dos trabalhadores da saúde foram os testes rápidos MedTeste Coronavírus (COVID-19) imunoglobulina G (IgG)/Imunoglobulina M (IgM), com Registro na Agência Nacional de Vigilância Sanitária sob número: 80560310056, na primeira semana do estudo. A testagem rápida foi utilizada no estudo entre o décimo e o décimo segundo dia de sintomas. Em sequência, frente às novas determinações para testagem, passou-se a utilizar o teste de Reação da Transcriptase Reversa, seguida da Reação em Cadeia da Polimerase (RT-PCR), disponibilizado na rede pública pelo Ministério da Saúde durante a pandemia. O RT-PCR foi coletado entre o quarto e o oitavo dias de sintomatologia dos trabalhadores. As testagens utilizadas no estudo foram coletadas uma vez em cada quadro sintomático do trabalhador. 

### Variáveis do estudo

As variáveis quantitativas foram idade, tempo de formação e dias de trabalho, que caracterizaram a amostra. Também, estratificou-se a amostra em dois grupos, o grupo dos trabalhadores que testaram negativo (GN) e outro composto pelos que testaram positivo (GP). O trabalhador da saúde que apresentou um resultado positivo, independentemente do número de testes realizados, foi incluído no grupo positivo. Logo, o grupo de negativos refere-se a não ter positivado em nenhum teste de monitoramento ao longo do período de estudo. 

As variáveis categóricas analisadas foram: a) dicotômicas (sim/não): doenças pulmonares, doenças cardiovasculares, recebimento de EPI, recebeu orientação sobre o uso de EPI, buscou informações, atendeu síndrome gripal, atendeu casos de COVID-19 e sexo (masculino/feminino); b) nominais: nível de formação (superior/médio) e acesso de pessoas ao setor de trabalho (limitado/restrito/assistência direto), como você considera seu conhecimento sobre os EPI (bom/excelente/mediano/muito ruim), qual o momento de maior risco para o trabalhador com relação à paramentação (ao vestir-se/ao remover as luvas para higienização das mãos/somente ao atender casos suspeitos/ao remover o avental/na retirada/outros), para essa variável os pesquisadores optaram por agrupar as respostas que possuíam baixa ocorrência na categoria “outros”. 

Também se analisaram o recebimento dos EPI e a disponibilidade de cada equipamento: touca, óculos de proteção, luvas, *face shield*, avental descartável, máscara N95 ou PFF, máscara cirúrgica e máscara de pano.

### Coleta de dados

A coleta dos dados ocorreu no período de maio a dezembro de 2020, realizadas até três tentativas de contato via chamada telefônica com os participantes para inclusão no estudo. Os pesquisadores encaminharam o formulário após os trabalhadores manifestarem os sintomas e confirmarem interesse em participar ou permanecer no estudo. 

Posteriormente ao contato, a realização do teste para COVID-19 ou coleta da amostra ocorreu nas dependências do ambulatório da policlínica municipal, ou nos centros de triagem montados para o enfrentamento da pandemia no município. O transporte e a análise das amostras foram realizados sob responsabilidade do Laboratório Central do Rio Grande do Sul (LACEN-RS). Mediante laudo emitido pelo LACEN para a Secretaria Municipal de Saúde, a equipe de pesquisa era comunicada e então eram realizados os contatos telefônicos. Durante a ligação telefônica, informava-se o resultado, após os pesquisadores forneciam orientações de cuidados pertinentes ao quadro apresentado por cada trabalhador da saúde e, após o aceito dele em participar da pesquisa, pactuava-se o envio do formulário. Destaca-se que a criação do formulário *online* e contato telefônico deram-se no intuito de promover a segurança e evitar sujeitar os indivíduos envolvidos no risco.

### Análise dos dados

Obtiveram-se as respostas aos formulários a partir do relatório de dados da nuvem em planilha *Excel Office* disponível pela plataforma utilizada. Revisou-se o preenchimento dos dados, sendo verificadas as perguntas de controle para a análise de qualidade dos dados. Um caso foi considerado perda do acompanhamento, visto que não finalizou o preenchimento do formulário na segunda testagem, restando na amostra apenas a sua primeira testagem e o formulário correspondente. Em seguida, os dados foram transportados para o *software Statistical Package for the Social Sciences (SPSS)* versão 25.0, no qual foi realizada uma análise descritiva, média e desvio-padrão (DP) (±).

Além disso, realizou-se a comparação de amostras independentes pelo teste de Qui-quadrado de Pearson ou Exato de Fisher, assumindo como a associação quando o p for menor de 0,05 e intervalos de confiança de 95%. 

### Aspectos éticos

O estudo foi aprovado pelo Comitê de Ética em Pesquisa sob o parecer n.º 30837420.0.0000.5323 e respeitou todos os preceitos éticos da Resolução 466/12 do Conselho Nacional de Saúde. Quanto ao Termo de Consentimento Livre e Esclarecido (TCLE), foi disponibilizado de forma *online*. Ressalta-se que, apenas após o participante concordar com a participação, selecionando a opção “Li e estou ciente da pesquisa e quero participar” no TCLE, o questionário ficava disponível para ser preenchido.

## Resultados

Dos 750 trabalhadores da saúde acompanhados, participaram desta pesquisa 206 que apresentaram sintomas gripais nos primeiros seis meses da pandemia, os quais correspondem a 27% do total de trabalhadores da saúde monitorados. Dentre os testados, 70 (34,0%) positivaram em algum momento durante os seis meses de acompanhamento. A média de idade foi de 39 anos completos (DP±11), sendo que a idade mínima foi de 19 anos e a máxima 81 anos. Já a média do tempo de formação foi de 10 anos (DP±9), sendo o tempo mínimo de formação um ano e máxima de 29 anos. Quanto às características da jornada de trabalho, os dias ativos na semana foram de cinco dias (DP±1) em média, com sete horas diárias (DP±3).

A maioria dos participantes acompanhados no estudo era do sexo feminino, 156 (75,72%) e 50 (24,28%) do sexo masculino, 182 (88,34%) sem problemas pulmonares e 164 (79,61%) sem alterações cardiovasculares; 143 (69,41%) eram trabalhadores de nível superior e 161 (78,15%) prestavam assistência direta aos pacientes. Na Tabela 1, estão dispostas as características dos trabalhadores da saúde, segundo resultado dos testes.

**Tabela 1:** Caracterização dos participantes segundo resultado dos testes para COVID-19 (n = 206). Uruguaiana, RS, Brasil, 2020

Variáveis	GN* 136 (66,0%)	GP^‡^ 70 (34,0%)
	N (%)^†^	N (%)^†^
**Sexo**		
Feminino	107 (78,7)	49 (70)
Masculino	29 (21,3)	21 (30)
**Doença pulmonar**		
Não	117 (86,0)	65 (92,9)
Sim	19 (14,0)	5 (07,1)
**Doença cardiovascular**		
Não	111 (81,6)	53 (75,7)
Sim	25 (18,4)	17 (24,3)
**Nível de escolaridade**		
Médio	41 (30,1)	22 (31,4)
Superior	95 (69,9)	48 (68,6)
**Trabalho em setor de**		
Acesso restrito	2 (1,5)	0 (0)
Acesso limitado	24 (17,6)	19 (27,1)
Assistência direta	110 (80,9)	51 (72,9)

^*^GN = Grupo dos Negativos; **‡**GP = Grupo dos Positivos; **†**Número absoluto e (%)

Identificou-se que, entre a totalidade de participantes, 197 (95,63%) trabalhadores receberam algum tipo de EPI, enquanto nove (4,37%) ficaram desassistidos em relação ao recebimento. Quanto as orientações para o uso desses equipamentos, 144 (69,90%) manifestaram ter recebido algum tipo de informação e 146 (70,87%) buscaram ativamente informações sobre a utilização deles. No que tange à percepção de conhecimento sobre o uso dos EPI, 152 (73,78%) manifestaram bom conhecimento, seguido de 27 (13,10%) excelente, 25 (12,13%) mediano e dois (0,97%) muito ruim.

Quando examinados os tipos de EPI que os participantes receberam, sete (3,39%) manifestaram ter recebido toucas, 12 (5,82%) óculos de proteção, 147 (71,35%) luvas, 145 (71,35%) *face shield*, 159 (77,18%) avental descartável, 140 (67,96%) máscara N95 ou PFF2, 162 (78,64%) máscara cirúrgica e 115 (55,82%) máscaras de pano. Cabe destacar que os trabalhadores da saúde receberam mais de um tipo de EPI.

Na análise dos atendimentos, verificou-se que 165 (80,9%) trabalhadores da saúde atenderam em algum momento indivíduos apresentando sintomas característicos de síndrome gripal e 173 (83,98%) afirmaram ter atendido indivíduos que testaram positivo para COVID-19. Assim, considerando os resultados supracitados, na Tabela 2, observa-se haver disponibilidade adequada dos EPI aos trabalhadores da saúde.

**Tabela 2:** Disponibilidade e conhecimento sobre EPI* dos trabalhadores de saúde na assistência a COVID-19 segundo resultado dos testes (n = 206). Uruguaiana, RS, Brasil, 2020

Variáveis	GN^†^ 136 (66,0%)	GP^§^ 70 (34,0%)	p^||^
	N (%)^‡^	N (%)^‡^	
Receberam EPI* ^¶^	132 (97,1)	65 (92,9)	0,963
Receberam orientação sobre o uso do EPI* ^¶^	94 (69,1)	50 (71,4)	0,134
**Buscaram informações** ^¶^	116 (85,3)	54 (77,1)	0,045
**Atenderam pacientes com síndrome gripal** ^¶^	110 (80,9)	55 (78,6)	0,024
A tenderam casos de COVID-19^¶^	112 (82,4)	61 (87,1)	0,138
**Risco de contágio na paramentação**			
Na retirada dos EPI*^¶^	75 (55,1)	45 (64,3)	0,084
Somente ao atender aos casos suspeitos^¶^	34 (25)	7 (10)	0,001
Outros^¶^	27 (19,9)	18 (25,7)	0,256
EPI* recebidos			
Touca^**^	4 (2,9)	3 (4,3)	0,265
Óculos de proteção^**^	8 (5,9)	4 (5,7)	1,256
Luvas^¶^	99 (72,8)	48 (68,6)	0,337
*Face shield* ^*¶*^	91 (66,9)	54 (77,1)	0,281
Avental descartável^¶^	103 (75,7)	56 (80)	0,212
Máscara N95^¶^	89 (65,4)	51 (72,9)	0,707
Máscara cirúrgica^¶^	113 (83,1)	49 (70)	0,003
Máscara de pano^¶^	72 (52,9)	43 (61,4)	0,897

^*^EPI = Equipamento de Proteção Individual; ^†^GN = Grupo dos Negativos; ^§^GP = Grupo dos Positivos; ^‡^N (%) = Número absoluto e (%); ^||^p = Nível de significância (p < 0,05); ^¶^Qui-quadrado de Pearson; ^**^Teste de Exato Fisher

Identificou-se associação estatística na análise de Qui-quadrado em relação ao resultado do teste e às variáveis de disponibilidade de máscara cirúrgica (p=0,003). E também, acerca das variáveis de conhecimento dos participantes sobre qual o momento de maior risco para o contágio da doença (p=0,001), em que houve predomínio dos trabalhadores que consideraram risco somente ao atender casos identificados como suspeitos para COVID-19. 

Também, notou-se associação com os participantes que mencionaram ter buscado informações sobre o uso correto dos EPI de forma autônoma (p=0,045). Ainda, ter atendido pessoas com sintomatologia característica de síndrome gripal foi associado à testagem positiva para COVID-19 (p=0,024). 

Quanto à classificação do conhecimento dos trabalhadores da saúde sobre os EPI, no grupo G1, 16 (11,8%) excelente, 99 (72,8%) bom, 19 (14%) mediano e 2 (1,5%) muito ruim. Já no G2, autoavaliaram-se como excelente 11 (15,7%), 53 (75,7%) bom, 6 (8,6%) mediano e nesse grupo nenhum participante manifestou um conhecimento muito ruim.

## Discussão

Os EPI são, inquestionavelmente, itens de segurança fundamentais para promover a proteção dos trabalhadores da saúde, visando minimizar a exposição direta ao vírus SARS-CoV-2. Conforme os dados deste estudo, nota-se que não houve totalidade de trabalhadores que receberam os EPI, tornando uma parcela desses trabalhadores da saúde ainda mais suscetíveis à infecção. 

Nessa diretiva, chama-se a atenção para a importância da preservação dos riscos ocupacionais que eles enfrentam e a obrigatoriedade das instituições em fornecer esses equipamentos, visto que é o primeiro passo para a garantia da saúde e segurança dessa população. Os trabalhadores da saúde foram considerados o recurso mais valioso de cada país, frente à indisponibilidade de reposição ágil de profissionais vivenciada no enfrentamento da pandemia^20^.

Com relação à discrepância, no que tange ao acesso aos EPI em cada país, principalmente quando analisada a disponibilidade para a APS, nota-se que em países com menor poder de mercado, assim como no Brasil, os trabalhadores da saúde vivenciaram problemas em relação à escassez de EPI e à baixa qualidade desses equipamentos. Por sua vez, países com maiores condições monetárias dispuseram de equipamentos extras de proteção, associados à alta tecnologia de engenharia, preservando a saúde da sua força de trabalho^21^. 

Comparando o recebimento de EPI entre os grupos que testaram positivo para COVID-19 e os que testaram negativo, identificou-se que o recebimento da máscara cirúrgica estava relacionado ao grupo dos testados negativos. Ou seja, acredita-se que receber e utilizá-la configura-se como um fator de extremo impacto para a diminuição do contágio da COVID-19, resultado que corrobora o estudo publicado anteriormente à pandemia da COVID-19, que considera as atualizações acerca da Síndrome Respiratória Aguda Grave (SARS). 

Assegura-se, portanto, a alta eficácia das máscaras cirúrgicas na redução da transmissão do vírus^22^ ^)-(^ ^23^.

Em outros estudos realizados com trabalhadores da saúde expõe-se que a utilização dos EPI durante a prática clínica configura-se como uma tarefa desafiadora e pode, ainda, ter efeitos nocivos para a saúde, tais como: disfunções neurológicas e físicas, manifestadas por cefaleia, irritabilidade, dificuldade na tomada de decisões, náuseas e dispneia^24^ ^)-(^ ^25^. Esses efeitos foram associados com reações orgânicas como a hipóxia e hipercapnia, fatores ambientais, como trabalhar em ambiente fechado, altas temperaturas, umidade e, também, fatores estressores causados pelo próprio momento pandêmico^24^ ^)-(^ ^25^.

Entretanto, é plausível reforçar que os benefícios superam os riscos, justificando a adesão corretamente quanto ao uso de EPI nas práticas laborais. A utilização da máscara pelos trabalhadores da saúde mostra-se de extrema relevância no estudo, reforçando a importância de ser implementada em todas as etapas da assistência aos pacientes que procuram os serviços de saúde manifestando sintomatologia compatível com a síndrome gripal, desde o momento da sua chegada, triagem, espera até sua alta/transferência ou óbito^26^. Tal comportamento deve superar o período da pandemia e ser considerado um protocolo cotidiano dos serviços de saúde no atendimento aos pacientes com sintomatologia respiratória compatível com doenças virais.

Ademais, faz-se necessário salientar que as máscaras de pano, embora tenham sido amplamente empregadas para proteção da população no início da pandemia, não são recomendadas para a utilização por trabalhadores da saúde; essas não se configuram como um EPI padrão em razão, principalmente, da inexistência de indicações de sua eficácia contra a transmissão do vírus SARS-CoV-2^23^. Ainda assim, o estudo promove como reflexão o número expressivo de trabalhadores da saúde que receberam a máscara de pano para utilizar em seu local de trabalho, dos quais 43 (61,4%) positivaram em algum momento para COVID-19 no período de acompanhamento.

Tem-se como hipótese que os fatores que levaram à opção da máscara de pano pelos trabalhadores da saúde foram a escassez de máscaras cirúrgicas, insuficientes para suprir a demanda de utilização no contexto de análise. Porém, as recomendações^27^ já alertavam que essa máscara deveria ser o último recurso utilizado na assistência de saúde a qualquer paciente, sendo ele sintomático respiratório ou não. Além disso, o órgão também orientava que, na ausência de máscaras N95 ou PFF2 para a troca no período indicado, o trabalhador deveria utilizá-las além do tempo de validade indicado pelos fabricantes, porém salientou-se que não havia garantia de eficácia nessas condições^27^. Esses fatos podem ter contribuído significativamente para a alta frequência de casos entre os trabalhadores da saúde que testaram positivo para COVID-19 no estudo.

Além da economicidade e durabilidade pelo seu reuso, outro fator que reforçou a utilização de máscaras de pano na população em geral foi a necessidade de priorização do insumo para os trabalhadores da saúde. Resultados de estudo americano que debateu a priorização desta oferta entre os trabalhadores da saúde mostraram que apenas 1/3 (33%) dos profissionais de enfermagem tinham acesso às máscaras N95, enquanto para a equipe médica totalizaram-se 100%^28^. 

Nesse sentido, ressalta-se que, para garantir a eficácia dos EPI, eles necessitam ser utilizados da forma correta e seguindo as indicações recomendadas pelos fabricantes. Assim, faz-se necessária a realização de capacitações e treinamentos frequentes aos trabalhadores da saúde, circunstância reiterada pelos dados desse estudo, nos quais os profissionais participantes que atenderam pessoas com sintomas de síndrome gripal testaram positivo para COVID-19 em algum momento do estudo (p=0,024). Ainda, um em cada cinco profissionais acreditava que o momento de maior risco de infecção era somente ao realizar atendimento aos usuários identificados como suspeitos. 

Assim, torna-se clara a importância de treinamentos quanto às questões de paramentação e desparamentação, tais como noções sobre a atenuação da doença e sobre o manejo de usuários suspeitos ou confirmados^29^. Estudos reiteram que trabalhadores que utilizam os EPI corretamente e estão atentos quanto ao tempo indicado de uso são menos suscetíveis à infecção pela COVID-19, aumentando a sua sensação de segurança^29^ ^)-(^ ^30^. 

Para mais, em consonância com as afirmações supracitadas, observa-se que os participantes do estudo que sinalizaram ter buscado por informações de forma autônoma acerca da utilização correta dos EPI contaminaram-se menos para COVID-19. Corroborando os achados, estudo revelou que o conhecimento acerca do manejo correto dos EPI está diretamente relacionado aos fatores de proteção^31^. 

A implementação de protocolos de atendimentos, bem como a realização de treinamentos para as equipes, é fundamental para ocorrer adesão aos cuidados referentes à paramentação correta e necessária, a fim de minimizar a ocorrência de infecções pelo SARS-CoV-2 em trabalhadores da saúde. Portanto, o uso foi indispensável durante a pandemia, sendo fundamental estruturar a logística de abastecimento destes insumos, além de organizar e implementar um planejamento que promova o uso adequado e racional desses EPI^32^, mesmo diante de casos esporádicos ou surtos pós-pandemia. 

Do mesmo modo, sugere-se que o alto índice de trabalhadores da saúde infectados possa ter sido influenciado pelo manejo ou momento inadequado na utilização, envolvendo a paramentação e desparamentação dos EPI. Isso foi constatado pelos 62 (30,1%) dos participantes que responderam não ter recebido orientações acerca da utilização correta dos equipamentos. Nesse ponto de vista, estudo comprova que quando o trabalhador da saúde utiliza o EPI e está bem orientado e atualizado em relação à sua finalidade, ele possui uma probabilidade menor de infectar-se pelo vírus SARS-CoV-2^10^. 

Deve-se ainda considerar que, no cenário pandêmico, muitos trabalhadores da saúde precisaram ser contratados ou realocados de suas funções rotineiras, visando suprir a necessidade dos serviços de saúde no enfrentamento de momentos críticos. Tal situação foi causada pelo alto número de pessoas e trabalhadores da saúde infectados, condições essas que exigiram um aprendizado imediato^10^ ^),(^ ^33^ ^)-(^ ^34^. Este fato reforça, novamente, a importância da implementação de instrumentos e capacitações no intuito de fornecer noções básicas da rotina dos serviços de saúde quanto à proteção laboral^33^.

Dentre as limitações do estudo, considerou-se não terem sido contemplados dados referentes à utilização dos EPI em tempo integral na rotina de trabalho ou à frequência de sua substituição pelo profissional e nem foi perguntado sobre a escassez de insumos em algum período. Outro aspecto que requer atenção é a utilização do teste rápido apenas na primeira semana do estudo, aplicado a nove participantes. A mudança ocorreu em razão da publicação de nota técnica estadual. Considerando o método de monitoramento, a diretriz de exclusividade do RT-PCR para testagem no estudo acompanhou a realidade do contexto em análise.

Menciona-se que os resultados encontrados por meio deste estudo no contexto da COVID-19 salientaram a necessidade da realização de pesquisas acerca do tema da segurança em saúde dos trabalhadores da APS no tocante à exposição às doenças infectocontagiosas, visto que a literatura científica enfatiza o enfrentamento em âmbito hospitalar. Além disso, deve-se considerar a elaboração de ações pertinentes para o campo da educação em saúde, a fim de nortear os trabalhadores da saúde a respeito da importância da utilização dos EPI em suas práticas laborais. Tão importante quanto prover os equipamentos, é que eles sejam utilizados da forma correta e indicada.

## Conclusão

O estudo identificou uma alta frequência de trabalhadores da saúde sintomáticos e positivos para COVID-19. Além disso, os resultados apontam claramente que a distribuição, utilização e orientação acerca do manejo correto dos EPI na prática assistencial dos trabalhadores da saúde é indispensável para a prevenção contra a COVID-19, com especial ênfase na utilização da máscara cirúrgica. 

Destarte, este estudo demonstrou uma fragilidade da gestão dos serviços ao presumir que os trabalhadores da saúde detêm conhecimentos suficientes acerca do uso de EPI no seu cotidiano de trabalho. Desse modo, não pode ser considerado suficiente o fornecimento dos EPI desarticuladamente da orientação assistencial para o uso dos mesmos. Com isso, os resultados indicaram a necessidade da valorização e orientação para a proteção efetiva dos trabalhadores de enfermagem e da APS.

# Translation: Infección y uso de equipos de protección individual entre trabajadores de la Atención Primaria de Salud durante la pandemia de COVID-19

**DOI:** 10.1590/1518-8345.6870.4289

**Authors:** Jarbas da Silva Ziani, Jenifer Härter, Bruna Lixinski Zuge, Debora da Cruz Payão Pellegrini, Josefine Busanello, Karlo Henrique dos Santos Herrera

**Affiliations:** 1 Universidade Federal de Santa Maria, Centro de Ciências da Saúde, Santa Maria, RS, Brasil.; 2 Universidade Federal do Pampa, Centro de Ciências da Saúde, Uruguaiana, RS, Brasil.; 3 Universidade Federal do Pampa, Centro de Ciências da Saúde Animal, Uruguaiana, RS, Brasil.

**Keywords:** Equipo de Protección Personal, Covid-19, Atención Primaria de Salud, Personal de Salud, Pandemias, Salud Laboral

## Abstract

**Objetivo:**

analizar la frecuencia y los factores de riesgo asociados a la infección por COVID-19 y la disponibilidad de Equipos de Protección Individual utilizados por los trabajadores de la atención primaria de salud.

**Método:**

estudio transversal, con duración de seis meses, realizado en Rio Grande do Sul. Para el análisis, se realizó análisis descriptivo, con comparación de muestras independientes mediante la prueba de Chi-cuadrado de Pearson y la prueba Exacta de Fisher (p<0,05).

**Resultados:**

participaron del estudio 206 (27%) trabajadores de la salud que presentaron síntomas de COVID-19. Se verificó asociación estadística para las variables disponibilidad de mascarilla quirúrgica (p=0,003), buscar información sobre el uso correcto del equipo de protección individual (p=0,045), haber atendido a personas con síndrome gripal (p=0,024) y creer que el mayor riesgo de contaminación es al atender a un paciente positivo a la enfermedad por coronavirus (p=0,001).

**Conclusión:**

la disponibilidad de equipos de protección individual es fundamental para prevenir la COVID-19, con especial énfasis en el uso de la mascarilla quirúrgica. Además, el estudio señaló la importancia de proporcionar Equipos de Protección Individual junto con orientaciones de cuidado para su uso.

## Destacados

**(1)** Señaló impactos en la distribución de EPI necesario para la seguridad del trabajador.

**(2)** Destacó la necesidad de capacitación y entrenamiento sobre el uso de EPI.

**(3)** Hubo significancia en cuanto a la disponibilidad de la mascarilla quirúrgica.

**(4)** Señaló la necesidad de realizar investigaciones sobre el tema de la seguridad en salud.

**(5)** Evidenció alta incidencia de trabajadores sintomáticos y positivos a COVID-19.

## Introducción

La pandemia de la enfermedad por coronavirus (COVID-19) se ha convertido en un tema emblemático para el mundo, ya que ha desafiado los sistemas de salud de los países afectados desde sus inicios^1^. En vista de su carácter de diseminación acelerada^1^ y de las estadísticas oficiales que resultaban alarmantes, datos actualizados mostraron que, en abril de 2023, el mundo ya contaba con 6.908.554 muertes, de las cuales 700.811 ocurrieron en suelo brasileño^2^.

Aunque todavía hay aparición de nuevas mutaciones y adaptaciones virales, se estima que una persona infectada con el *Severe Acute Respiratory Syndrome Coronavirus* 2 (SARS-CoV-2) tiene la posibilidad de contagiar a entre 1,5 y 3,5 personas más^3^. Los trabajadores de la salud tienen hasta tres veces más probabilidades de infectarse en comparación con la población general, ya que, en su rutina laboral, están constantemente expuestos al contacto con pacientes infectados por el virus^4^ ^)-(^ ^5^. 

Al analizar la cantidad de casos registrados a nivel mundial, se observa que el 15% representa el contagio en trabajadores de la salud^6^. En julio de 2021, durante el pico de la pandemia en Brasil, hubo 120.240 (27,1%) casos de trabajadores de la salud infectados por el virus y 549 (96,2%) óbitos, lo que representa una muerte cada 17 horas en ese período^7^. Ante este escenario, la Atención Primaria de Salud (APS) resultó esencial en el combate a la pandemia, atendiendo hasta el 80% de todos los casos sintomáticos leves y moderados^8^.

Los Equipos de Salud de la Familia (ESF), que operan en sus territorios, juegan un papel fundamental en la red asistencial de cuidados, siendo esenciales para combatir cualquier epidemia^9^. Por lo tanto, para que la atención se realizara de manera segura, fue necesario el uso de Equipos de Protección Individual (EPI) durante la asistencia. Un estudio demostró que el 85% de los trabajadores de la salud que utilizaron EPI durante su atención, especialmente la mascarilla N95, no contrajeron COVID-19^10^. 

En este sentido, se destaca el deber de las instituciones de salud de dotar a sus trabajadores de los EPI necesarios, así como brindar las orientaciones adecuadas para su correcto uso^11^. Dicho equipo sirve como barrera mecánica individual contra los patógenos, minimizando la exposición de los trabajadores de la salud. Los más utilizados para la atención de pacientes con COVID-19 fueron mascarillas, guantes, delantales desechables, gorros, batas, protectores faciales, gafas protectoras y respirador para partículas del tipo NIOSH-*certified* (N95, N99 y N100) y las Piezas Faciales Filtrantes (PFF1, PFF2 o PFF3)^11^. 

En los últimos años, el campo de la salud y de la seguridad del trabajador ha establecido medidas de intervención encaminadas a promover protección, promoción, recuperación y rehabilitación de la salud y de la seguridad del trabajador, por medio de la creación de políticas, programas y prácticas que busquen el bienestar^12^. La salud de estos trabajadores enfrentó riesgos laborales de morbimortalidad sin precedentes, relacionados con aspectos considerados nocivos para su seguridad, causados principalmente por la falta de EPI asociada a la exposición a pacientes infectados, sobrecarga de trabajo y falta de control de infecciones^13^.

Como lo demuestra un estudio coreano, hubo un aumento significativo en la carga de trabajo de los trabajadores de la salud, ya que ellos, además de brindar cuidados y asistencia específicos a los pacientes infectados, también comenzaron a realizar la limpieza de los ambientes. Esto, sumado a la escasez de trabajadores, suministros médicos y EPI, ha resultado en malas condiciones laborales para estos trabajadores de la salud^14^. 

En varios momentos durante la pandemia de COVID-19, los países se vieron afectados por escasez en la cadena de producción y suministro global, altos precios y problemas con la calidad de los productos, así como se obstaculizó la entrega oportuna de materiales, lo que dificultó que las instituciones de salud pudieran mantener *stock* para garantizar la protección de los trabajadores de la salud^15^. En este sentido, se destaca el papel fundamental del gobierno en esta situación, ya que le corresponde coordinar esfuerzos para garantizar que las zonas geográficas más afectadas cuenten con el equipamiento necesario^15^. 

Ante esto, el presente estudio se justifica por la necesidad de reflexiones e investigaciones sobre la calidad del trabajo de los trabajadores de la APS expuestos a los cambios provocados por la pandemia de COVID-19, ya que se evidencian estudios centrados predominantemente en aspectos relacionados con los trabajadores que actúan en la atención hospitalaria. Además, es importante resaltar que es de suma importancia que estos trabajadores reciban mayor visibilidad, pues son quienes constituyen la primera línea de la APS, que se caracteriza por ser la principal puerta de entrada y triaje de pacientes sintomáticos de COVID-19 en los servicios de salud, de cara a apaciguar la pandemia. 

Los resultados del estudio pueden ayudar a los trabajadores de la salud, especialmente al enfermero, con información para la práctica en salud y enfermería, permitiendo reflexionar sobre la necesidad de revisión constante de los protocolos de bioseguridad y uso de equipos de protección individual en los cuidados rutinarios en salud. Por lo tanto, el objetivo de este manuscrito fue analizar la frecuencia y los factores de riesgo asociados a la infección por COVID-19 y la disponibilidad de Equipos de Protección Individual utilizados por los trabajadores de la APS.

## Método

### Diseño del estudio

Se trata de un estudio transversal, realizado con trabajadores de la salud. En la organización del texto, se siguieron las recomendaciones de las guías *Checklist for Reporting Results of Internet E-Surveys* y *Strengthening the Reporting of Observational Studies in Epidemiology* (STROBE) para estudios transversales^16^.

### Lugar del estudio

El estudio se realizó en el municipio de Uruguaiana, Rio Grande do Sul-RS, Brasil. La ciudad tiene un importante interés estratégico comercial e internacional, considerando que se encuentra equidistante de las capitales Porto Alegre, Montevideo, Buenos Aires y Asunción^17^. En cuanto al número de casos de COVID-19 la ciudad registra, hasta el momento, 155.362 infectados con la enfermedad, y de ellos, 637 fallecidos^18^.

La cobertura de salud del municipio por equipos de la Atención Primaria se estima en 66%, ya que cuenta con 19 unidades de salud con 23 ESF en el perímetro urbano y un Equipo de Salud Familiar Rural. También cuenta con un equipo de salud itinerante, que atiende a usuarios de zonas rurales del municipio que no están cubiertas por las unidades de salud locales^19^.

### Población del estudio

La población del estudio estuvo compuesta por todos los trabajadores de la salud (médicos, enfermeros, técnicos y auxiliares de enfermería, dentistas, farmacéuticos, auxiliares de farmacia, fisioterapeutas, nutricionistas, fonoaudiólogos, biomédicos, servicio social, educador físico, veterinarios, psicólogos, personal administrativo, higienistas y recepcionistas), todos vinculados a la Secretaría Municipal de Salud (N=750), quienes fueron monitoreados durante seis meses, de marzo a agosto de 2020. No se realizó ningún cálculo de muestra, ya que se incluyeron todos los empleados para el seguimiento y, en ese momento, no había estudios que estimaran la proporción de trabajadores de la salud sintomáticos.

Como criterio de inclusión, se consideró a los trabajadores que estuvieron activos durante la pandemia de COVID-19. En cuanto al criterio de exclusión, se consideró los trabajadores que fueron hospitalizados por síntomas antes de la fecha prevista para la realización de las pruebas en la Secretaría Municipal de Salud, imposibilitados de responder el cuestionario. 

Los trabajadores fueron monitoreados por el estudio y considerados casos sospechosos de COVID-19, con indicación de prueba diagnóstica, al presentar síntomas y signos clínicos compatibles con síndrome gripal, como fiebre, tos, secreción nasal, dolor de garganta, dolor de cabeza, vómitos, diarrea, disnea y mal estado general. También fueron testeados y evaluados si compartían el espacio de trabajo con otro profesional sintomático que presentó resultado positivo, o si se ausentaban de las actividades laborales por síntomas, mediante notificación por *Short Message Service* (SMS) o aislamiento domiciliario por recomendación clínica, con indicación de prueba para COVID-19 por solicitud médica. Por lo tanto, se incluyeron en el estudio 206 trabajadores de la salud que en algún momento durante el período de seguimiento presentaron síntomas característicos de la COVID-19.

El seguimiento se realizó mediante contacto telefónico, realizado tras la notificación de síntomas vía SMS. Los trabajadores de la salud recibieron un cuestionario después de la primera prueba, independientemente del resultado. Durante el seguimiento, con cada nuevo episodio de síntomas reportados por los trabajadores, se realizaron repeticiones de pruebas de COVID-19 y para las pruebas posteriores a la primera solamente en los casos de resultado positivo se recolectaron nuevamente los datos por medio del formulario. 

### Instrumento de medida

El formulario aplicado consistió en un bloque de preguntas en su mayoría cerradas y buscó recopilar información pertinente a las características demográficas, socioeconómicas, síntomas de gripe y momento de aparición de los mismos. También contenía información sobre lugar de trabajo, número de horas de atención a pacientes, conocimiento, uso y disponibilidad de EPI. El formulario fue creado por los autores de la investigación, con base en la literatura científica y en sus cuestionamientos sobre el tema. Después de su creación, el formulario fue enviado a un comité de cinco expertos en salud para evaluar el contenido. Luego de los ajustes recomendados, se definió la versión final siguiendo todas las sugerencias. 

Posteriormente, se creó un formulario *on line* en la plataforma *Google Forms*, con el objetivo de facilitar el acceso a los participantes, especialmente durante un período de pandemia, ya que se puede acceder desde cualquier computadora y dispositivos móviles, abarcando todos los sistemas operativos una vez que haya acceso a Internet. Para la creación del formulario se tuvo en cuenta una encuesta rápida realizada a los gestores de servicios sobre la disponibilidad de estos recursos por parte de los trabajadores de la salud. 

A continuación, el formulario fue sometido a una prueba piloto con 15 miembros del grupo de investigación que eran trabajadores de la salud, excluidos de la muestra analizada. El propósito del piloto fue analizar si los ítems eran fáciles de entender y aptos para ser respondidos, por medio de una consulta *on line*.

Las pruebas utilizadas para detección de COVID-19 en los trabajadores de la salud fueron las pruebas rápidas MedTeste Coronavirus (COVID-19) inmunoglobulina G (IgG)/Inmunoglobulina M (IgM), registradas en la *Agência Nacional de Vigilância Sanitária* con el número: 80560310056, en la primera semana del estudio. Se utilizaron pruebas rápidas entre el décimo y el duodécimo día de los síntomas. Posteriormente, ante las nuevas determinaciones para las pruebas, se utilizó la prueba de Reacción de Transcriptasa Inversa seguida de la prueba de Reacción en Cadena de la Polimerasa (RT-PCR), puesta a disposición en la red pública por el *Ministério da Saúde* durante la pandemia. La RT-PCR se recogió entre el cuarto y el octavo día de los síntomas de los trabajadores. Las pruebas utilizadas en el estudio se recolectaron una vez para cada condición sintomática del trabajador. 

### Variables del estudio

Las variables cuantitativas fueron edad, tiempo de formación y días de trabajo, que caracterizaron la muestra. Asimismo, la muestra se estratificó en dos grupos, el grupo de trabajadores que dieron negativo (GN) y otro compuesto por los que dieron positivo (GP). El trabajador de la salud que dio positivo, independientemente del número de pruebas realizadas, se incluyó en el grupo positivo. Por tanto, el grupo negativo hace referencia a no haber dado positivo en ninguna prueba de seguimiento durante todo el periodo de estudio. 

Las variables categóricas analizadas fueron: a) dicotómicas (sí/no): enfermedades pulmonares, enfermedades cardiovasculares, recibimiento de EPI, recibió orientación sobre el uso de EPI, buscó información, atendió síndrome gripal, atendió casos de COVID-19; y género (masculino/femenino); b) nominales: nivel de formación (superior/medio) y acceso de las personas al sector laboral (limitado/restringido/asistencia directa), ¿cómo considera usted su conocimiento sobre EPI (bueno/excelente/regular/muy malo)?, cual es el momento de mayor riesgo para el trabajador con relación a la paramentación (al vestirse/al quitarse los guantes para lavarse las manos/solamente al tratar casos sospechosos/al quitarse el delantal/al quitarse/otros) - para esta variable, los investigadores optaron por agrupar las respuestas que tuvieron baja ocurrencia en la categoría “otros”. 

También se analizó el recibimiento de los EPI y la disponibilidad de cada equipo: gorro, gafas protectoras, guantes, *face shield*, delantal desechable, mascarilla N95 o PFF, mascarilla quirúrgica y mascarilla de tela.

### Recolección de datos

La recolección de datos se llevó a cabo de mayo a diciembre de 2020, y se realizaron hasta tres intentos de contactar a los participantes mediante llamada telefónica para su inclusión en el estudio. Los investigadores enviaron el formulario después de que los trabajadores manifestaron síntomas y confirmaron su interés en participar o permanecer en el estudio. 

Tras el contacto, la prueba de COVID-19 o la toma de muestra se realizó en las instalaciones ambulatorias del policlínico municipal o en los centros de triaje habilitados para combatir la pandemia en el municipio. El transporte y el análisis de las muestras fueron realizados bajo la responsabilidad del *Laboratório Central do Rio Grande do Sul* (LACEN-RS). A través de un informe emitido por el LACEN a la Secretaría Municipal de Salud, se notificaba al equipo de investigación y luego se realizaban los contactos telefónicos. Durante la llamada telefónica se informaba el resultado, luego los investigadores brindaban orientaciones de cuidados pertinentes a la situación presentada por cada trabajador de la salud y, después de aceptar participar de la investigación, se acordaba enviar el formulario. Es de destacar que la creación del formulario *on line* y el contacto telefónico se realizaron con el objetivo de promover la seguridad y evitar exponer a riesgos a las personas involucradas.

### Análisis de los datos

Las respuestas a los formularios se obtuvieron del reporte de datos de la nube en una hoja de cálculo de *Excel Office* disponible en la plataforma utilizada. Los datos completados fueron revisados y se verificaron las preguntas de control para el análisis de la calidad de los mismos. Un caso se consideró perdido del seguimiento, ya que el formulario no se completó durante la segunda prueba, quedando solamente la primera prueba y el formulario correspondiente en la muestra. Luego, los datos fueron transportados al *software Statistical Package for the Social Sciences* (SPSS) versión 25.0, en el cual se realizó un análisis descriptivo, media y desviación estándar (DE) (±).

Además, se compararon muestras independientes mediante la prueba de Chi-cuadrado de Pearson o la prueba Exacta de Fisher, asumiendo asociación cuando p es menor que 0,05 e intervalos de confianza del 95%. 

### Aspectos éticos

El estudio fue aprobado por el Comité de Ética en Investigación bajo dictamen n° 30837420.0.0000.5323 y respetó todos los preceptos éticos de la Resolución 466/12 del Consejo Nacional de Salud. En cuanto al Formulario de Consentimiento Libre e Informado (TCLE, por sus siglas en portugués), se puso a disposición *on line*. Cabe señalar que, solamente después de que el participante aceptó participar, seleccionando la opción “He leído y tengo conocimiento de la investigación y quiero participar” en el TCLE, el cuestionario quedaba disponible para ser completado.

## Resultados

De los 750 trabajadores de la salud acompañados, participaron en esta investigación 206 que presentaron síntomas gripales en los primeros seis meses de la pandemia, lo que corresponde al 27% del total de trabajadores de la salud monitoreados. Entre los examinados, 70 (34,0%) dieron positivo en algún momento durante los seis meses de seguimiento. La edad promedio fue de 39 años (DE±11), siendo la edad mínima 19 años y la edad máxima 81 años. El tiempo medio de formación fue de 10 años (DE±9), siendo el tiempo mínimo de formación de un año y el máximo de 29 años. En cuanto a las características de la jornada laboral, los días activos en la semana fueron cinco días (DE±1) en promedio, con siete horas diarias (DE±3).

La mayoría de los participantes monitoreados en el estudio fueron mujeres, 156 (75,72%), y 50 (24,28%) hombres, 182 (88,34%) sin problemas pulmonares y 164 (79,61%) sin alteraciones cardiovasculares; 143 (69,41%) eran trabajadores con educación superior y 161 (78,15%) brindaban atención directa a los pacientes. En la Tabla 1 se presentan las características de los trabajadores de la salud, según los resultados de las pruebas.

**Tabla 1:** Caracterización de los participantes según los resultados de las pruebas de COVID-19 (n = 206). Uruguaiana, RS, Brasil, 2020

Variables	GN* 136 (66,0%)	GP^‡^ 70 (34,0%
	N (%)^†^	N (%)^†^
**Género**		
Femenino	107 (78,7)	49 (70)
Masculino	29 (21,3)	21 (30)
**Enfermedad pulmonar**		
No	117 (86,0)	65 (92,9)
Sí	19 (14,0)	5 (07,1)
**Enfermedad cardiovascular**		
No	111 (81,6)	53 (75,7)
Sí	25 (18,4)	17 (24,3)
**Nivel de escolaridad**		
Medio	41 (30,1)	22 (31,4)
Superior	95 (69,9)	48 (68,6)
**Trabajo en sector de**		
Acceso restringido	2 (1,5)	0 (0)
Acceso limitado	24 (17,6)	19 (27,1)
Asistencia directa	110 (80,9)	51 (72,9)

*GN = Grupo de los Negativos; **‡**GP = Grupo de los Positivos; **†**Número absoluto y (%)

Se identificó que, entre el total de participantes, 197 (95,63%) trabajadores recibieron algún tipo de EPI, mientras que nueve (4,37%) se encontraban sin asistencia en cuanto a su recepción. Respecto a las pautas sobre el uso de estos equipos, 144 (69,90%) afirmaron haber recibido algún tipo de información y 146 (70,87%) buscaron activamente información sobre su uso. En cuanto a la percepción del conocimiento sobre el uso de los EPI, 152 (73,78%) manifestaron tener buen conocimiento, seguidos de 27 (13,10%) excelente, 25 (12,13%) regular y dos (0,97%) muy malo.

Al examinar los tipos de EPI que recibieron los participantes, siete (3,39%) reportaron haber recibido gorros, 12 (5,82%) gafas protectoras, 147 (71,35%) guantes, 145 (71,35%) *face shield*, 159 (77,18%) delantal desechable, 140 (67,96%) mascarilla N95 o PFF2, 162 (78,64%) mascarilla quirúrgica y 115 (55,82%) mascarillas de tela. Cabe señalar que los trabajadores de la salud recibieron más de un tipo de EPI.

En el análisis de la atención, se verificó que 165 (80,9%) trabajadores de la salud habían atendido en algún momento a personas que presentaban síntomas característicos del síndrome gripal y 173 (83,98%) afirmaron haber atendido a personas que dieron positivo a la prueba de COVID-19. Así, considerando los resultados mencionados anteriormente, en la Tabla 2 se observa que existe una adecuada disponibilidad de EPI para los trabajadores de la salud.

**Tabla 2:** Disponibilidad y conocimiento sobre EPI* de los trabajadores de la salud en atención de COVID-19 según resultado de las pruebas (n = 206). Uruguaiana, RS, Brasil, 2020

Variables	GN^†^ 136 (66,0%)	GP^§^ 70 (34,0%)	p^||^
	N (%)^‡^	N (%)^‡^	
Recibieron EPI* ^¶^	132 (97,1)	65 (92,9)	0,963
Recibieron orientación sobre el uso del EPI* ^¶^	94 (69,1)	50 (71,4)	0,134
**Buscaron información** ^¶^	116 (85,3)	54 (77,1)	0,045
**Atendieron a pacientes con síndrome gripal** ^¶^	110 (80,9)	55 (78,6)	0,024
**Atendieron casos de COVID-19** ^¶^	112 (82,4)	61 (87,1)	0,138
Riesgo de contagio en la paramentación			
Al quitarse los EPI*^¶^	75 (55,1)	45 (64,3)	0,084
Solamente al atender casos sospechosos^¶^	34 (25)	7 (10)	0,001
Otros^¶^	27 (19,9)	18 (25,7)	0,256
EPI* recibidos			
Gorro**	4 (2,9)	3 (4,3)	0,265
Gafas protectoras**	8 (5,9)	4 (5,7)	1,256
Guantes^¶^	99 (72,8)	48 (68,6)	0,337
*Face shield* ^*¶*^	91 (66,9)	54 (77,1)	0,281
Delantal desechable^¶^	103 (75,7)	56 (80)	0,212
Mascarilla N95^¶^	89 (65,4)	51 (72,9)	0,707
Mascarilla quirúrgica^¶^	113 (83,1)	49 (70)	0,003
Mascarilla de tela^¶^	72 (52,9)	43 (61,4)	0,897

*EPI = Equipo de Protección Individual; ^†^GN = Grupo de los Negativos; ^§^GP = Grupo de los Positivos; ^‡^N (%) = Número absoluto y (%); ^||^p = Nivel de significancia (p < 0,05); ^¶^Chi-cuadrado de Pearson; ^**^Prueba Exacta de Fisher

Se identificó asociación estadística en el análisis de Chi-cuadrado respecto del resultado de la prueba y las variables de disponibilidad de mascarilla quirúrgica (p=0,003). Y también sobre las variables de conocimiento de los participantes sobre cuál es el momento de mayor riesgo para el contagio de la enfermedad (p=0,001), en que predominaron los trabajadores que consideraron riesgo solamente al atender casos identificados como sospechosos de COVID-19. 

También se observó asociación con los participantes que mencionaron haber buscado información sobre el uso correcto de los EPI de forma autónoma (p=0,045). Además, haber atendido a personas con síntomas característicos del síndrome gripal se asoció con dar positivo a COVID-19 (p=0,024). 

En cuanto a la clasificación del conocimiento de los trabajadores de la salud sobre los EPI, en el grupo G1, 16 (11,8%) excelente, 99 (72,8%) bueno, 19 (14%) regular y 2 (1,5%) muy malo. En el G2, 11 (15,7%) se autoevaluaron como excelente, 53 (75,7%) bueno, 6 (8,6%) regular, y en este grupo ningún participante manifestó conocimiento muy malo. 

## Discusión

Los EPI son, sin duda, elementos de seguridad fundamentales para promover la protección de los trabajadores de la salud, con el objetivo de minimizar la exposición directa al virus SARS-CoV-2. Según los datos de este estudio, se observa que no todos los trabajadores recibieron EPI, lo que hace que una parte de estos trabajadores de la salud sea aún más susceptible a la infección. 

En esta directiva se llama la atención sobre la importancia de preservar los riesgos laborales que enfrentan y la obligación de las instituciones de proporcionar estos equipos, por ser el primer paso para garantizar la salud y la seguridad de esta población, ya que se consideraba a los trabajadores de la salud como el recurso más valioso de cada país, ante la falta de disponibilidad de reposición ágil de profesionales vivida en el enfrentamiento a la pandemia^20^.

En cuanto a la discrepancia en el acceso a los EPI en cada país, especialmente al analizar la disponibilidad para la APS, se observa que en los países con menor poder de mercado, así como en Brasil, los trabajadores de la salud experimentaron problemas relacionados con la escasez de EPI y la baja calidad de estos equipos; mientras tanto, los países con mayores condiciones monetarias contaron con equipos de protección extra, asociados a alta tecnología de ingeniería, preservando la salud de su fuerza laboral^21^. 

Al comparar el recibimiento de EPI entre los grupos que dieron positivo a COVID-19 y los que dieron negativo, se identificó que la recepción de la mascarilla quirúrgica estaba relacionada con el grupo que dio negativo. Es decir, se cree que recibirlo y utilizarlo es un factor de extremo impacto en la reducción de la propagación de la COVID-19, resultado que corrobora el estudio publicado previo a la pandemia de COVID-19, que toma en cuenta las actualizaciones sobre el Síndrome Respiratorio Agudo Severo (SARS, por sus siglas en inglés).

Por tanto, la alta eficacia de las mascarillas quirúrgicas para reducir la transmisión del virus está asegurada^22^ ^)-(^ ^23^.

Otros estudios realizados con trabajadores de la salud muestran que el uso de EPI durante la práctica clínica es una tarea desafiante y, también, puede tener efectos nocivos para la salud, como disfunciones neurológicas y físicas, manifestadas por dolor de cabeza, irritabilidad, dificultad para tomar decisiones, náuseas y disnea^24^ ^)-(^ ^25^. Estos efectos estuvieron asociados a reacciones orgánicas como hipoxia e hipercapnia, factores ambientales como trabajar en espacio cerrado, altas temperaturas y humedad y, también, factores estresantes provocados por la propia pandemia^24^ ^)-(^ ^25^.

Sin embargo, es posible reforzar que los beneficios superan los riesgos, lo que justifica la correcta adherencia al uso de EPI en las prácticas laborales. El uso de mascarillas por parte de los trabajadores de la salud se revela de suma importancia en el estudio, reforzando la importancia de su implementación en todas las etapas de la atención de los pacientes que acuden a los servicios de salud y presentan síntomas compatibles con el síndrome gripal, desde el momento de su llegada, triaje, espera, hasta el alta/transferencia u óbito^26^. Tal comportamiento debe incluso sobrevivir al período pandémico y ser considerado un protocolo diario de los servicios de salud en la atención a pacientes con síntomas respiratorios compatibles con enfermedades virales.

Además, es necesario destacar que las mascarillas de tela, si bien fueron muy utilizadas para proteger a la población al inicio de la pandemia, no están recomendadas para los trabajadores de la salud, ya que no constituyen EPI estándar debido, principalmente, a la falta de indicios de su eficacia contra la transmisión del virus SARS-CoV-2^23^. Aun así, el estudio promueve la reflexión sobre el importante número de trabajadores de la salud que recibieron mascarilla de tela para usar en su lugar de trabajo, de los cuales 43 (61,4%) dieron positivo a COVID-19 en algún momento del período de seguimiento.

Se plantea la hipótesis de que los factores que llevaron a la elección de mascarilla de tela por parte de los trabajadores de la salud fue la escasez de mascarillas quirúrgicas, que fueron insuficientes para satisfacer la demanda de uso en el contexto de análisis. Sin embargo, las recomendaciones^27^ ya advertían que esta mascarilla debería ser el último recurso en asistencia sanitaria para cualquier paciente, con o sin síntomas respiratorios. Además, el organismo también orientaba que, a falta de mascarillas N95 o PFF2 para el cambio en el período recomendado, el trabajador debería utilizarlas más allá de la fecha de vencimiento indicada por los fabricantes, aunque se señaló que no había garantía de efectividad bajo estas condiciones^27^. Estos hechos pueden haber contribuido pueden haber contribuido significativamente a la alta frecuencia de casos entre los trabajadores de la salud que dieron positivo a COVID-19 en el estudio.

Además de la economía y durabilidad por su reutilización, otro factor que reforzó el uso de mascarillas de tela en la población general fue la necesidad de priorizar el insumo para los trabajadores de la salud. Los resultados de un estudio estadounidense que debatió la priorización de esta oferta entre los trabajadores de la salud mostraron que solamente 1/3 (33%) de los profesionales de enfermería tenían acceso a mascarillas N95, mientras que para el equipo médico era del 100%^28^. 

En este sentido, es importante destacar que para garantizar la eficacia de los EPI es necesario utilizarlos correctamente y siguiendo las instrucciones recomendadas por los fabricantes. Por ello, es necesario realizar frecuentes capacitaciones y entrenamientos a los trabajadores de la salud, circunstancia que reiteran los datos de este estudio, en el que los profesionales participantes que atendieron a personas con síntomas de síndrome gripal dieron positivo a COVID-19 en algún momento del estudio (p=0,024). Además, uno de cada cinco profesionales creía que el momento de mayor riesgo de contagio era solamente al atender a los usuarios identificados como sospechosos.

Así, queda clara la importancia de la capacitación en temas de paramentación y desparamentación, como nociones sobre mitigación de la enfermedad y manejo de usuarios sospechosos o confirmados^29^. Estudios reiteran que los trabajadores que utilizan correctamente los EPI y prestan atención al período de uso recomendado son menos susceptibles a la infección por COVID-19, lo que aumenta su sensación de seguridad^29^ ^)-(^ ^30^. 

Además, en línea con las afirmaciones antes mencionadas, se observa que los participantes del estudio que indicaron haber buscado de forma autónoma información sobre el uso correcto de los EPI se infectaron menos con COVID-19. Corroborando los hallazgos, un estudio reveló que el conocimiento sobre el correcto manejo de los EPI está directamente relacionado con los factores de protección^31^. 

La implementación de protocolos de atención, así como la capacitación de los equipos, es fundamental para el cumplimiento de los cuidados relacionados con la paramentación correcta y necesaria, con el fin de minimizar la ocurrencia de infecciones por SARS-CoV-2 en los trabajadores de la salud. Por lo tanto, su uso fue esencial durante la pandemia, siendo fundamental estructurar la logística de suministro de estos insumos, además de organizar e implementar una planificación que promueva el uso adecuado y racional de estos EPI^32^, incluso ante casos esporádicos o brotes post pandemia. 

Asimismo, se sugiere que la alta tasa de trabajadores de la salud infectados puede haber sido influenciada por el manejo o momento inadecuado de uso, que involucra la paramentación y la desparamentación de los EPI. Así lo confirmaron 62 (30,1%) de los participantes que respondieron que no habían recibido orientación sobre el uso correcto de los equipos. Desde este punto de vista, un estudio demuestra que cuando el trabajador de la salud utiliza el EPI y está bien orientado y actualizado sobre su finalidad, tiene menos probabilidad de infectarse con el virus SARS-CoV-2^10^.

También se debe considerar que, en el escenario pandémico, muchos trabajadores de la salud tuvieron que ser contratados o reubicados de sus funciones rutinarias, buscando cubrir la necesidad de los servicios de salud ante momentos críticos, provocada por el alto número de personas y trabajadores de la salud infectados, condiciones que requirieron aprendizaje inmediato^10^ ^),(^ ^33^ ^)-(^ ^34^. Este hecho refuerza, una vez más, la importancia de implementar instrumentos y capacitaciones para brindar nociones básicas de la rutina de los servicios de salud en materia de protección laboral^33^. 

Entre las limitaciones del estudio, se consideró que no se incluyeron datos sobre el uso de EPI a tiempo completo en la rutina de trabajo ni la frecuencia de reemplazo de estos por parte del profesional, tampoco se preguntó sobre la escasez de insumos en algún período. Otro aspecto que requiere atención es el uso de la prueba rápida solamente en la primera semana del estudio, aplicada a nueve participantes. El cambio se produjo debido a la publicación de una nota técnica estatal. Considerando el método de seguimiento, la pauta de exclusividad de RT-PCR para las pruebas en el estudio siguió la realidad del contexto analizado.

Se menciona que los resultados encontrados por medio de este estudio en el contexto de COVID-19 resaltaron la necesidad de realizar investigaciones sobre el tema de seguridad en salud de los trabajadores de la APS en términos de exposición a enfermedades infecciosas, dado que la literatura científica enfatiza el afrontamiento en el ámbito hospitalario. Además de desarrollar acciones relevantes para el campo de la educación en salud, con el fin de orientar a los trabajadores de la salud sobre la importancia del uso de los EPI en sus prácticas laborales, ya que, tan importante como proporcionar los equipos, es que sean utilizados de la forma correcta e indicada.

## Conclusión

El estudio identificó una alta frecuencia de trabajadores de la salud sintomáticos y positivos a COVID-19. Además, los resultados indican claramente que la distribución, uso y orientación sobre el correcto manejo de los EPI en la práctica asistencial de los trabajadores de la salud es fundamental para la prevención de la COVID-19, con especial énfasis en el uso de la mascarilla quirúrgica. 

Por lo tanto, este estudio demostró una debilidad en la gestión de los servicios al asumir que los trabajadores de la salud tienen conocimientos suficientes sobre el uso de EPI en su trabajo diario. De este modo, no puede considerarse suficiente la provisión de EPI de forma desconectada de la orientación asistencial para su uso. Por ende, los resultados indicaron la necesidad de valoración y orientación para la protección efectiva de los trabajadores de enfermería y de la APS.
